# The Effects of Diet, Dietary Supplements, Drugs and Exercise on Physical, Diagnostic Values of Urine Characteristics

**DOI:** 10.3390/nu16183141

**Published:** 2024-09-17

**Authors:** Dorota Skrajnowska, Barbara Bobrowska-Korczak

**Affiliations:** Department of Toxicology and Food Science, Faculty of Pharmacy, Medical University of Warsaw, Banacha 1 Street, 02-091 Warsaw, Poland; dorota.skrajnowska@wum.edu.pl

**Keywords:** diet, dietary supplements, drugs, urinalysis

## Abstract

**Background/Objectives:** This review summarizes the current knowledge about factors that affect the physical characteristics of urine. It highlights proper urine sample collection and displays factors like diet, hydration status, and medications that can alter urine color, odor, clarity, specific gravity and pH. **Results:** Urinalysis is a minimally invasive examination of a patient’s health, especially concerning nephrological and endocrinological abnormalities, as well as dietary habits and stimulants used. Certain deviations in appearance, composition or frequency/pain during urination may indicate an ongoing disease process in the body. Based on laboratory results, further medical treatment is determined. The reason for a change in the color of the urine, for its clouding or intense odor may be a disease, as well as the consumption of food, medication, intensive physical exercise or inadequate hydration of the body. Well-standardized procedures for collecting, transporting, preparing and analyzing samples should become the basis for an effective diagnostic strategy in urinalysis. It is worth noting that pharmacists in pharmaceutical care are often the first people to whom a patient turns for health advice and for the interpretation of simple laboratory tests. Acquiring the ability to interpret the results of laboratory tests and the principles of proper sampling for laboratory tests is indispensable in the process of possible counseling and providing reliable answers to patients’ questions. **Conclusions:** Although urinalysis is not recommended as a routine screening tool for the general population, it can prove to be a valuable source of patient health data in some cases as the data will be useful to physicians and pharmacists to more effectively diagnose and better care for patients.

## 1. Introduction

There are many physiological processes in the body that result in the production of unnecessary, and sometimes toxic, metabolic products, which are mainly removed by the kidneys. The end product of the multistage purification of the blood is urine, which is 95% water; the remainder is numerous compounds of both organic and inorganic structures. Urinalysis is the most basic examination and provides a great deal of information about a patient’s state of health [1,2]. Based on the laboratory results, further medical treatment is determined. Urinalysis is a minimally invasive examination of a patient’s health especially concerning nephrological and endocrinological abnormalities, as well as dietary habits and stimulants used. The reason for a change in the color of the urine, for its clouding or intense odor may be a disease, as well as the consumption of food, medication, intensive physical exercise or inadequate hydration of the body [2,3,4,5,6,7,8,9,10,11,12], for example, taking vitamins B2 and B12 may result in a bright yellow color of urine [13,14], while supplements containing tryptophan cause a purple color of urine [15]. Excessive supplementation with preparations of iron or black licorice may increase the risk of black-colored urine [16]. A red color is most often indicative of hematuria but can also result from the consumption of foods containing red pigments (e.g., beetroot, berries, blackberries, products containing carotene) [17] or from the use of certain drugs (e.g., nitrofurantoin, metronidazole, rifampicin) [18,19,20]. Red-colored urine, darkening after exposure to sunlight, is also characteristic of an acute porphyria attack [21]. Dehydration will constrict the components of the urine and alter its transparency, specific gravity and pH [6,9,22]. On the other hand, overhydration, taking stimulant drugs or the presence of certain cancers, as well as liver, kidney and adrenal diseases, hypothyroidism, hemolytic jaundice, diabetes mellitus and hypoproteinemia can result in colorless urine [11,23,24,25,26,27,28,29]. There are a number of foods (including asparagus, garlic brassica vegetables, fish and meat proteins, eggs, alcohol, coffee and beans) the consumption of which in some individuals may result in a specific odor, generally associated with the presence of characteristic metabolites [12,30,31,32,33,34,35]. Urine odor is also strongly influenced by diseases such as diabetes mellitus [36]; bacterial and fungal infections [37]; liver, intestinal and pancreatic diseases [34,38]; and genetic diseases such as trimethylaminaemia, phenylketonuria, maple syrup disease or isovaleric acidosis [39,40,41,42,43,44]. A prerequisite for the reliability of the results obtained is the observance of basic sampling principles and awareness of the influence of a number of external factors on the final results. 

Well-standardized procedures for collecting, transporting, preparing and analyzing samples should become the basis for an effective diagnostic strategy in urinalysis. The proper collection of the sample guarantees the correct interpretation of the results by the doctor [45,46,47,48,49,50]. 

This review summarizes the current knowledge about factors that affect the physical characteristics of urine. It highlights proper urine sample collection and displays factors like diet, hydration status and medications that can alter urine color, odor, clarity, specific gravity and pH. 

## 2. Proper Collection of Urine Sample for Analysis 

Following basic rules during urine collection is very important to obtain diagnostically reliable results [45,46]. During urinalysis, the first morning sample is generally used, because it has the highest diagnostic value; it is also the best for microbiological examination, i.e., urine culture [47,48,49]. The composition of urine is variable and depends on a great many factors, such as the amounts and types of fluids ingested, food consumed and stimulants or drugs taken. In order to minimize the variability in the composition of urine samples, it is most common to test urine from the first morning micturition, after a prior toilet, from the middle stream (50–150 mL). However, there are cases in which a physician or laboratory diagnostician consulting to prepare for the test may recommend collecting the first stream of urine (e.g., diagnosis of *Chlamydia trachomatis*) [50]. It is worth noting that with the use of newer nucleic acid amplification techniques (NAATs) and greater sensitivity, the timing of sample collection in a *C. trachomatis* test may not be as important as thought [51]. The conditions of collection are very important, as the urethra is colonized by bacteria and may contain numerous leukocytes. Therefore, it is necessary to perform a morning toilet to remove various secretions from its outlet, for example, from the prostate in men and the reproductive tract in women [47,48]. Maintaining this basic hygiene will lend credibility to the test results. A typical result indicative of inadequate collection is high titers of bacteria and flat epithelial cells in the absence of leukocytes [47,48,52]. 

Another option that is usually used for emergency testing or when the patient is unable to collect the first sample is a second urine sample, given 2 to 4 h after the first morning sample [53]. Finally, the so-called random urine sample collected at any time of the day applies primarily to young children and newborns due to the lack of large gaps between micturitions and the poor ability to concentrate the urine [54]. Pouches with hypoallergenic adhesive tape are most commonly used. The genital and anal areas are washed and then the pouch is placed by pressing the adhesive tape against the perineum; the contents are checked every 15 min [54,55]. In any case, the description of the sample delivered to the laboratory should include information about the type of sample collection. 

A slightly different type of collection is the daily urine collection (AWD) [56,57]. It is best to use a special graduated container into which each portion of urine should be collected throughout a 24 h period. If possible, it should be stored in a dark and cool place. The rules for collecting such a sample are a little different, namely, the first morning portion of urine should be completely transferred to the toilet and the collection should begin with the second morning urine sample, with the starting time being recorded. Each subsequent portion of urine should be passed in its entirety (without dividing it into initial, middle and final streams) for 24 h from the starting hour. Thus, the last portion of urine collected into the container is the first morning urine sample (omitted when starting the collection) [58,59,60]. At the end of the collection, read the volume of urine collected, mix the whole well (without foaming), and then transfer 50–100 mL into a standard urine container, signed and named, with information about the volume of total urine and the time of the start and end of collection.

In addition to the proper collection of a urine sample, it is extremely important to follow some basic rules before analyzing the urine:

– For a minimum of 24 h before urine collection for laboratory testing, avoid hard work or extreme exercise [52,61] and a high-protein diet [62,63].
– At least one day before the scheduled urine collection for laboratory testing, it is recommended to maintain sexual abstinence [64]. Examination of urine after sexual intercourse can be difficult due to the possible presence of a large number of sperm in the urine, which prevents accurate microscopic evaluation of the urine sediment. There may also be minor damage to the urethra, which will result in the presence of increased epithelium, red blood cells or the presence of bacteria in the urine.
– Collecting urine for laboratory tests during monthly bleeding and 2–3 days before and after menstruation is not recommended (unless otherwise instructed by the referring physician) [65]. When samples are collected during menstruation, the urine is often contaminated with a large number of red blood cells and epithelium, making it difficult to obtain reliable results. This also imposes interpretations in the direction of hematuria and suspicion of, for example, nephrolithiasis or nephrotic syndrome.
– The urine container should be a disposable plastic one, specially purchased for this purpose; if the sample is for microbiological testing, the container should be sterile [52].
– The container with the collected urine should go to the collection point in the shortest time possible (up to a maximum of 2 h at room temperature, or, as a last resort, 4 h at 4 °C because delays can affect the test results, e.g., change in pH, bacterial growth, decomposition of morphotic elements) [52,66]. However, it should be noted that there are opinions that keeping a container of urine in the refrigerator is allowed only if this material is intended for microbiological examination (culture), and it should not last longer than two hours.
– If possible, diuretics should be discontinued, as they affect the amount of urine excreted, as well as changes in electrolyte levels—sodium (Na), potassium (K), calcium (Ca) and others [67]. It is necessary to remember to balance the effect of diuretics, that is, to provide adequate amounts of fluids on a regular basis because otherwise, dehydration can occur. The normal volume of excreted urine is 1000–2500 mL/24 h. The minimum amount of urine needed to excrete the products of protein metabolism with an average supply of protein and dietary salt and the full capacity of the kidneys to thicken the urine is 400–500 mL/day [52,68].

## 3. The Effects of Diet, Dietary Supplements, Drugs and Exercise on the Physical and Diagnostic Values of Urine Characteristics

The parameters to be evaluated during urinalysis can be divided into the following:

– Physical characteristics: color, odor, transparency, specific gravity and pH [48,52,69].
– Chemical features: protein, glucose, urobilinogen, ketone bodies, bilirubin, uric acid, nitrogenous compounds and others [48,52,69].
– Microscopic analysis of urine [7,70,71] necessary for proper diagnosis in many asymptomatic cases, including urinary tract infections, urinary tract tumors, latent glomerulonephritis or interstitial nephritis. The urine sediment is mainly white and red blood cells, epithelia, rollers, lipids, bacteria, mucus, fungi, parasites and minerals. The result is generally given as a total from 10 fields of view. Microscopic examination of the urine sediment is an integral part of the total urine examination [70,71]. During the final interpretation of the obtained result of the urine sediment analysis, it is necessary to take into account all other parameters, as well as the patient’s clinical symptoms. Mineral compounds may be present in the urine sediment in the form of crystals or amorphous precipitates. These include uric acid crystals, amorphous urates and calcium oxalates. These compounds are often formed in urine that has been stored in a refrigerator prior to testing. It should be noted that manual microscopic examination of urine is time-consuming and requires considerable experience for results interpretation; therefore, in modern laboratories, this process has been automated using flow cytometry and digital microscopy [70,71].

The remainder of this article will discuss the various physical parameters of urine that may be affected by diet/fluids/physical activity/health conditions/medications.

### 3.1. Changes in the Color of Urine

Normal urine has a straw-yellow, clear color, which indicates adequate hydration of the body. The darker the shade, the greater the likelihood that there is too little water in the body. A change in urine color is an easily noticeable parameter and often worries patients. However, many of the causes of abnormal urine color are benign effects of medications and foods (recently eaten foods and the natural or synthetic dyes they contain) and taking certain medications. Sometimes a change in urine color can also be a sign of illness. If the change in urine color persists for several days, you should see a specialist, have a general urine test, a blood test and, if necessary, a urinary ultrasound.

In general, the color of urine depends on the following:

– The presence of dyes produced by endogenous metabolism, such as urochrome, uroerythrin, urobilinogen [72,73].
– The degree of urine thickening resulting from various causes, discussed in detail later in this article [72].
– Diet (e.g., beets, berries, rhubarb, broad beans, fava beans, aloe vera, carrots, green spinach, purine-rich foods, excess protein, laxatives) [13,14,15,74,75,76,77].
– Drug therapy or reagents used, e.g., chloroquine [13,78], nitrofurantoin [18], phenazopyridine [79], vitamins B [13,14], phenacetin [80], phenytoin [81], methylene blue [82], iron [13,16], methyldopa, rifampicin [83], isoniazid [13], warfarin [13], triamterene [84,85], phenolphthalein [86], phenothiazine [87], sulfasalazine [88], amitriptyline [84,85,89,90], propofol, indigo blue, indigocarmine, cimetidine, indomethacin, doxorubicin, promethazine, rinsapine, sildenafil [84,85,91,92], metronidazole [19,93], sorbitol [13,94], cresol, nitrates [95], tryptophan [96], hydroxycobalamin [97], acetaminophen [98], metoclopramide [90], herbicides [99] and cefozoppran [100].
– Metabolic disorders, e.g., hemolytic anemia, porphyrias [21], liver diseases, cancers [101], alkaptonuria [102], blue diaper syndrome, hypercalcemia, glucose-6-phosphate dehydrogenase deficiency, sickle cell anemia [13] and Lesch-Nyhan disease [103].
– Bacteremia, inflammation in the urinary tract [104,105,106,107].

#### 3.1.1. Dark Yellow/Amber-Colored Urine

May indicate dehydration due to inadequate fluid intake, working or being in a hot room or place [22], heavy physical exercise [108,109,110,111], severe diarrhea, use of laxatives, persistent vomiting or impaired kidney function [55,73,112,113,114,115,116]. This phenomenon is particularly dangerous in children and the elderly. Symptoms of dehydration such as vomiting, diarrhea, sunken fontanel and eyes, irritability and not having a wet diaper for more than 3 h occur in newborns and infants [117]. This poses an immediate life-threatening risk, so medical attention should be sought immediately [118,119,120]. On the other hand, elderly patients become dehydrated as a result of diarrhea, renal disease (including simple uremia and chronic renal failure), frequent constipation, use of laxatives and edema-reducing and hypotensive drugs [121,122,123,124]. Regardless of age, one must replenish fluids to prevent dehydration and reduce the risk of kidney stone formation. The optimal amount is 2–3 L of water per day, depending on gender, physical activity, health status and weather conditions [125]. 

#### 3.1.2. Colorless Urine

It may be a cause of excessive fluid retention or may occur as a result of the use of certain medications, particularly those that stimulate the kidneys to excrete more urine (diuretics, amitriptyline, tiotropium, cyclophosphamide, vincristine and sulfonylureas). Other causes include glycosides, glipizide, certain cancers (duodenal cancer, pancreatic cancer, thymoma), renal and adrenal disease, hypothyroidism, hemolytic jaundice, diabetes, hypoproteinemia and excessive vasopressin [23,24,25,26,27,28]. However, in most cases, the lack of urine staining usually indicates that one is simply drinking too much water at once (1.5–2 L) [29]. This becomes dangerous when the kidney’s filtration threshold is exceeded. The result is the accumulation of too much water in the bloodstream, which can upset the fluid balance, excessively dilute concentrations of endogenous compounds and flush out minerals and vitamins taken from the diet. Excess water in the intravascular and interstitial spaces can result in edema of peripheral tissues and lungs, hypertension and increased excretion of sodium and water ions by the kidneys. 

Excessive accumulation of water in the extracellular space can also result from a variety of diseases and be of a different nature [22,27,126,127,128,129,130], including the following:

– Isotonic—due to excessive supply of isotonic solutions such as sodium chloride in people with impaired renal function, the presence of cardiovascular insufficiency, liver disease, endocrine disorders (Cushing’s syndrome) and glucocorticosteroid treatment, there are increased amounts of water and sodium in the body [130].
– Hypertonic—there is also an excessive amount of sodium in the body, but water retention occurs secondary to the existence of an excessive amount of sodium in the blood [131,132,133]. Causes include excessive supply of hypertonic solutions by parenteral route, renal impairment due to chronic kidney disease or acute renal failure, excessive secretion of antidiuretic hormones (e.g., condition after extensive surgery) and increased secretion of glucocorticoids and mineralocorticoids (shock, adrenal hyperfunction). Hypertonic conductance can also occur in survivors who drink seawater containing a large amount of sodium ions.
– Hypotonic is also referred to as water intoxication, yet another water disorder proceeding with hyponatremia, i.e., a decrease in blood sodium concentration. This can occur as a result of excessive supply of electrolyte-deprived or electrolyte-deficient fluids (e.g., glucose solution) [126,127]. Hypotonic conductance can occur in patients with impaired renal water excretion or in patients with excessive antidiuretic hormone (ADH) secretion, in the course of porphyria, cranial trauma, inflammatory conditions of the brain and lungs (tuberculosis and pulmonary aspergillosis), in certain malignancies (oat cell carcinoma, duodenal and pancreatic cancer, thymoma) and in patients taking such drugs as sulfonylurea derivatives, carbamazepine, amitriptyline, thioridazine, diuretics, cyclophosphamide and vincristine [27,128,129,130,134].

Acute water intoxication can occur independently of the disease, for example, as a result of athletes drinking large amounts of electrolyte-free fluids, e.g., marathon runners, cyclists (up to 5 L) or infants fed very dilute milk mixtures (sodium deficiency) [26,135,136]. Nausea, vomiting, anorexia, pupil irregularity, muscle spasms, seizures and cerebral edema can then occur. If immediate specialized treatment is not given, then damage to the central nervous system, acidosis and even death can occur [137].

#### 3.1.3. Bright Yellow (Fluorescent) Coloration of Urine

Taking B vitamins, especially B2 (riboflavin) and B12 (cyanocobalamin), and numerous multivitamin supplements [13,14]. Very yellow-colored urine after vitamin supplementation is a normal condition and should not cause concern. The color is more intense than usual and often has a bright “canary” hue. Under UV light, a fluorescent effect can be seen [138]. However, if the intense coloration persists after vitamin withdrawal, a doctor should be consulted.

#### 3.1.4. Reddish-Pink/Red Coloration of Urine

Regardless of the intensity, such a color raises concern and its cause should be quickly detected, i.e., the presence of red blood cells or hemoglobin should be confirmed. If blood is indeed present, it can indicate various disorders ranging from kidney function, hematopoietic system or menstrual blood contamination. Red color can include terms such as pink colors, shades of red, orange, brown or black depending on the analyst examining the sample. 

The causes of staining with different shades of red can include the following:

– Presence of urate (gout) [139], inflammation of the urinary tract of various etiologies with excessive secretion of uric acid [140]. Hyperuricemia is the excessive accumulation of uric acid due to impaired metabolism of purines. Purines are a group of aromatic organic compounds that occur naturally in foods, especially those rich in protein, or from de novo synthesis and the breakdown of endogenous nucleic acids. Purines are converted into uric acid via metabolic pathways. Excess uric acid is deposited, among other things, in the joints, forming microcrystals and causing gout in addition to facilitating the crystallization of calcium oxalates (urolithiasis). Therefore, in the case of red-orange coloration of urine, and especially in the presence of crystals, hyperuricemia should be considered [139,140]. Some contradictions exist regarding the use of vitamin C as a prophylaxis for gout [141,142,143]. The beneficial effect of ascorbic acid on lowering serum uric acid levels is probably due to its uricosuric effect and inhibition of uric acid synthesis. Taking vitamin C in doses of about 500 mg per day may have a positive effect on lowering uric acid concentrations in healthy individuals [141,142,143]. However, for patients diagnosed with gout, vitamin C supplementation (without medication) has no significant effect [144]. On the other hand, vitamin C is a precursor of oxalic acid, and consumption of high doses (more than 1.500 mg/day), for a prolonged period of time will increase the risk of side effects, especially the risk of kidney stones, so the decision to implement supplementation should be carefully considered [145]. It is believed, however, that this does not apply to natural sources of vitamin C but precisely to dietary supplements.
– Consumption of foods rich in natural and added food dyes (beets, beet leaves, blackberries, blueberries, rhubarb, carrots) [17,74,75,76,94,146]. In this case, the most commonly described symptom is beeturia [17], which is the discoloration of urine after consuming raw, cooked or pickled beets, beet juice or foods enriched with beet extract. The typical color can range from pink to deep red, and the phenomenon occurs in 10–14% of the population, with increased frequency among those with iron deficiency or malabsorption syndrome. The pigments found in beets belong to betacyanins, hence the term betacyaniuria is sometimes used. It is worth noting that the compounds contained in beet (betanin, betalains, betacyanins), after extraction, are used as natural dyes in many foodstuffs such as carrot, orange and tomato fruit juices, tomato concentrates, ketchup, tomato soup, red gelatin, red and purple candies, yogurts, ice cream, icing, sweet fillings for baked goods and sausage products (salami, sausages), as well as in cheeses with additives. The occurrence of red, reddish-purple or even brownish urine as a result of consuming these products is harmless and transient. Similar urine color can be caused by the consumption of rhubarb. The change in urine color in this case, too, is the result of natural dyes found in the edible stalks, which survive the digestion stage in the stomach. Rhubarb stalks also contain small amounts of oxalic acid, which probably protects the pigment from digestive juices and thus contributes to the change in urine color [75,76].
– Accidental contamination with menstrual blood [147].
– Diseases resulting in the presence of free hemoglobin, erythrocytes, myoglobin and porphyrins in urine [13,21,148]. Hemoglobinuria is the excretion in the urine, hemoglobin being the oxygen carrier in the erythrocyte. After the breakdown of red blood cells, a trace amount of free hemoglobin appears in the blood [149]. However, in hemolytic anemia, if massive erythrocyte disintegration occurs, the amount of free hemoglobin increases significantly and, having exceeded the transport capacity of haptoglobin, it begins to appear in the urine and can darken it to a deep reddish color [150,151]. On the other hand, paroxysmal nocturnal hemoglobinuria (PNH, paroxysmal nocturnal hemoglobinuria) is an acquired clonal disease of the hematopoietic stem cell [148,152]. The disease was first described in 1882 in a 29-year-old laborer complaining of fatigue, abdominal pain and severe nocturnal attacks of hemoglobinuria, which worsened after excessive alcohol consumption, after exercise and after administration of iron salts.

Even without the presence of blood, dark red urine can be a sign of a serious hereditary condition, e.g., porphyria or abnormality of heme metabolism [21]. It manifests as dark urine, abdominal pain, photosensitive rashes or neuropsychiatric complaints, among other symptoms. The disease is difficult to detect because it is rare, and sometimes there is no equipment for urine porphyrin analysis, which further delays treatment [21]. Myoglobinuria is the excretion of myoglobin in the urine, which occurs in the course of rhabdomyolysis (damage to striated muscle) [153,154]. Myoglobin is a protein found in striated muscles—skeletal and in the heart—resembling in structure the hemoglobin present in red blood cells. Myoglobin’s primary function is to store oxygen. In the case of significant exertion, when the molecular pressure of oxygen reaches low values in the muscles, myoglobin releases the oxygen molecules attached to it, allowing the muscles to continue working efficiently using high-energy ATP. Only myoglobin released from damaged skeletal muscle and heart cells is found in the bloodstream. 

Myoglobin can also enter the urine, impairing kidney function and causing acute kidney failure.

– The use of drugs such as doxorubicin [155], daunorubicin [156], aminophenazone, phenazopyridine [79], quinine [157], chloroquinone [78], L-dopa, hydroquinone [158], naphthol [159], phenytoin, rifampicin [20], metronidazole [19], nitrofurantoin [18], phenacetin [87], phenothiazine [88], salazopyrin [18], barbiturates, lidocaine, diclofenac, clindamycin, erythromycin, clemastine, lutein, progesterone, gestagens, heparin [160] and hydroxycobalamin [97]. In toxicology practice, hydroxycobalamin is sometimes used to treat cyanide poisoning [161]. Traditional treatments rely on drugs such as amyl nitrite, sodium nitrite and sodium thiosulfate; these can cause methemoglobinemia, further reducing the ability of red blood cells to carry oxygen. Hydroxocobalamin works by combining with cyanide to form cyanocobalamin. An unintended but mild effect of its administration is a red tint to the skin and urine. This effect usually passes after a few days [97].
– The cause of red-colored urine may be lead or mercury poisoning, possibly related to kidney damage [162,163].
– Finally, the deliberate addition of blood or dye to a urine sample. Patients with psychiatric disorders add blood or another red substance directly to the sample being analyzed. The non-specific complaints they describe lead to numerous, usually fruitless tests. Simulation is difficult to diagnose and may require repeat urine samples obtained under observation to ultimately discover the primary disorder [164].

#### 3.1.5. Brown Coloration of Urine 

Brown or very dark red urine, possibly stored longer, is interpreted as brown and may result from the following:

– Eating larger amounts of rhubarb, broad beans and fava beans [77]. Consumption of foods prepared especially with fava beans changes the color of urine in some people. This is due to fava bean deficiency, or glucose-6-phosphate dehydrogenase deficiency, a congenital disease that leads to the breakdown of red blood cells [165]. Once broken down, the red blood cells release their contents into the blood and eventually into the urine, resulting in dark-colored urine. In people with favaism, eating fava beans triggers a chain of events that leads to blood in the urine and other complications (abdominal pain, vomiting [75].
– Diseases associated with excessive excretion of hemoglobin, myoglobin and porphyrin (described earlier) [13,21,148,153].
– Copper poisoning due to, among other things, hemoglobinuria [166].
– Dehydration, [22] including as a result of strenuous exercise [108,109,110,111].
– Liver diseases (cirrhosis, inflammation). Transparent dark brown urine can be a symptom of jaundice. This is even more certain if at the same time, the stool is discolored and the skin is yellowed [167].
– Cancer metastasis [101]. Brown urine can be a sign of melanocytes in the urinary tract. Metastatic melanoma can lead to a rare condition called diffuse melanosis, causing dark skin, distant internal organ changes and brown or black urine [168].
– Extremely tough exercises [108,109,111,153] during which muscle and/or kidney damage can occur.
– Acetaminophen overdose (increase in *p*-aminophenol). In the 1980s, it was observed that an overdose of acetaminophen (obtained using an older method of production) in addition to liver failure can cause the appearance of brown urine due to the accumulation of the metabolite *p*-aminophenol [169].

#### 3.1.6. Black Coloration of Urine

– Excessive supplementation with iron preparations, including in the form of injections, can exacerbate the risk of black urine [16,170]. Patients in this case simply need reassurance that this is a mild side effect of the drug.
– Use of laxatives like cascara (the peel that surrounds coffee beans) or *Folium Sennae* [171].
– The presence of melanin, which may appear if a cancer producing it (melanoma, melanoblastoma) grows in the body. The presence of melanin in the urine can cause brown discoloration, possibly with a black tinge [168].
– Presence of methemoglobin, for example, as a result of poisoning with nitrates (III/V) or aniline dyes [170,172].
– Presence of homogentisic acid. Alkaptonuria is a rare inherited disorder in which the body has an impaired ability to catabolize tyrosine and phenylalanine leading to the accumulation of homogentisine acid in the body [173]. Clinically, it manifests as arthritis and darkening of the skin and urine. Diagnosis is based on measuring the concentration of homogentisinic acid in the urine. There is no effective pharmacotherapy: high doses of vitamin C and a low-protein diet are recommended [173].
– The use of drugs such as aminophenazone, aminopyrine, antipyrine and quinine [157], chloroquine [13,78], hydroquinone, phenytoin, metronidazole [19,93], nitrates [172], nitrofurantoin [18], phenacetin [80], phenolphthalein [86], phenothiazine [87], salazopyrin, sorbitol and L-dopa α-Methyldopa (the latter two drugs can stimulate melanin synthesis and urinary excretion, a known side effect) [158,174]. Also worth mentioning is cresol, a disinfectant that can sometimes be consumed by alcohol addicts [175].
– Consumption of black licorice in large quantities can temporarily change the color of both stool and urine (from a dark green or almost black color, lasting several days) [75].

#### 3.1.7. Green–Blue Coloration of Urine 

Such unusual toying of the urine is most often the result of a certain diet or consumption of products containing synthetic dyes. However, this staining or intermediate staining can also appear after taking certain medications or indicate an infection caused by hepatitis or cancer. Below are a few examples:

– Foods rich in artificial dyes and foods like spinach and green asparagus. However, in some people, eating (especially) asparagus changes the color of urine to slightly green; unfortunately, the smell of urine also changes (ammoniacal) [75]. Carotene pigments are responsible for the color of the urine, while sulfur compounds are responsible for the odor.
– Taking certain medications can cause blue or green urine. For example amitriptyline, doxorubicin, indomethacin, cimetidine, metoclopramide, guaiacol, promethazine, triamterene, rinsapine, propofol, sildenafil and B vitamins [84,85]. The main reason for this phenomenon is the contact of metabolites present in the urine with air and their oxidation to blue–green compounds. This symptom regarding the above-mentioned drugs is mild and not subject to further evaluation when the other results of urinalysis are normal. Other compounds that can cause abnormal urine color are the supplement arbutin (from *Arctostaphylos uva-ursi*) and methylene blue, used for the treatment of methemoglobinemia, among others. Methemoglobinemia is a hematological disease in which a significant portion of hemoglobin from Fe (II) is replaced by methemoglobin from Fe (III) so that the ability to release oxygen to the tissues is reduced. Cyanosis, shortness of breath, fatigue, headaches and dizziness follow; in extreme cases, it can lead to coma or death. The treatment of methemoglobinemia was one of the first and most important uses of methylene blue (BM) [176,177]. In this case, however, the dose is key—while low doses of BM treat methemoglobinemia, high doses can provoke it (as intravenous injections). It is worth remembering that the use of high doses can also affect the green–blue staining of mucous membranes and urine. The problem is much smaller and virtually unnoticeable when using microdoses, i.e., <100 µg per day [92]. Methylene blue has weak antiseptic properties and is an ingredient in several drugs (such as Uroblue tablets) used to reduce symptoms of bladder inflammation or irritation [85] and as an antifungal, antibacterial and antimalarial drug [85,178,179]. Phosphasal, used for dysuria, also contains methylene blue, which causes blue–green coloration of the urine [85]. Methylene blue is increasingly used for oral mucositis in patients receiving anticancer therapy and sonodynamic effects [180,181]. Blue–green urine is a normal side effect of these drugs and is harmless.
– Dyes (e.g., methylene blue, indigocarmine) used intravenously in the diagnosis of certain kidney and bladder diseases can change the color of patients’ urine temporarily to blue [182,183].

However, the occurrence of green urine may be due to specific medical conditions:

– Blue diaper syndrome (Drummond syndrome) is a rare metabolic disorder that leads to impaired tryptophan absorption. This amino acid is degraded under the influence of bacteria in the intestinal lumen to indole dyes, which are then excreted by the kidneys and, upon contact with air, are oxidized, turning the urine blue. The characteristic symptom is just the presence of blue urine spots on the diaper of a sick infant. Other symptoms are hypercalcemia and nephrocalcinosis. Treatment is dietary restriction (limitation of protein, calcium and vitamin D supply) and antibiotic therapy [184].
– A rare genetic disorder is familial hypercalcemia. Children with this condition urinate blue urine from birth [185].
– Prostatitis, bladder bacteremia caused by urinary tract infections, mainly infection with *Pseudomonas aeruginosa*. In this case, the history and physical examination indicate an infectious disease, and urine and blood cultures will confirm the diagnosis. Treatment focuses on clearing the infection, not the color of the urine [89,186].

#### 3.1.8. Orange/Red–Orange Coloration of Urine

– Eating lots of raw carrots, drinking carrot juice, taking vitamin supplements (beta-carotene) [94].
– Use of laxatives (fluid loss occurs), such as those containing *Folium Sennae* [146].
– Excessive bilirubin secretion (in liver disorders) [13].
– The use of drugs such as phenazopyridine [187], rifampicin and high-dose riboflavin [146].

It is also worth mentioning two rare cases of the unexpected appearance of orange-colored urine:

– Accumulation of *Citrobacter sedlakii* bacteria [188] and colonization of the urinary tract and infection in neonates and immunocompromised patients. *Citobacter sedlakii* are Gram-negative bacilli of the *Enterobacteriaceae* family that produce orange indole by degrading tryptophan.
– The second case was associated with uric acid crystalluria [139]. It involved a patient diagnosed with epilepsy. Epileptic seizures can lead to hyperuricemia and renal failure, but orange-colored urine immediately after a seizure has not been reported. Sustained muscle contractions during epileptic seizures cause excessive ATP breakdown and as a result of the breakdown of purine nucleotides, uric acid concentrations increase. The likely causes of uric acid crystallization are acidic urine pH and high uric acid concentration. Thus, uric acid crystalluria should be considered in the differential diagnosis of any patient with reddish-orange colored urine, especially in the presence of macroscopic red–orange crystals [139].

#### 3.1.9. Milky/White Coloration of Urine

It can result from the following:

– Electrolyte disorders resulting in excess calcium and phosphate [189]. Amorphous phosphates in urine are a physiological component of urine that is alkaline or slightly acidic (in shape they resemble fine white-gray sand or in compact form they form putative rollers). Very abundant phosphates detected in urinalysis can be due to a number of causes, from an excessive supply of phosphate in the diet to phosphate lithiasis (calcium phosphates or ammonium–magnesium phosphates (struvite) appear, accompanied by an increase in the specific gravity of the urine) or urinary tract infection (in which case bacteria, erythrocytes and leukocytes may also appear). Phosphate lithiasis is a rare variety of renal deposits accompanying urinary tract infections. The bacteria responsible for the infection alkalize the urine, which promotes the precipitation of phosphate stones. Most often, however, kidney stones are caused by several factors simultaneously.
– Urinary tract infections (purulent infections with *E. Coli* species, also of the genus Proteus) [190,191]. Severe urinary tract infections can contribute to the appearance of white-colored urine, as purulent fluid can enter the bladder. In addition, the condition of so-called sterile leukocyturia (the presence of purulent fluid in the patient, but without the presence of bacteria), which is associated with urinary tract tuberculosis, should also be considered [192].
– Chyluria (the presence of chyle in the urine) [8]. It results from a malformation of the lymphatic system or is caused by obstruction of the lymphatic system caused by tumor growths or closure of the system by parasites (mainly pinworms). Also, poisoning by mosquito-borne nematodes (filariasis) causes abnormal communication of the lymphatic system with the urinary tract [193]. Another cause can also be a lymphatic fistula [194].
– Sometimes it can be caused by the presence of uric acid crystals from purine-rich foods (such as anchovies, herring and red meat) [195].

#### 3.1.10. Purple Coloration of Urine

– The so-called purple urinary pouch syndrome (PUBS) occurs in patients in post-obstructive states or in severe intestinal failure in patients with catheter insertion [196,197]. It indicates bacterial infection (most often by Providencia stuartti and rettgeri, Proteus mirabilis, Pseudomonas auruginosa, Klebsiella pneumoniae, Escherichia coli, Morganella and Citrobacter species, Enterococci and group B Streptococci) and the presence of tryptophan. Tryptophan in the digestive tract is converted to indoxyl, which is metabolized in the liver and excreted by the kidneys. Bacteria in the urine form indigo and indirubin, which together cause purple urine, staining the Foley catheter (made of polyvinyl chloride) [198]. Generally, purple-stained urine is associated with Gram-negative bacteriuria and usually resolves with antibiotic treatment and catheter change.
– Increased dietary tryptophan content is increased substrate availability for conversion [15]. Protein-rich foods include fish (tuna and cod), cheeses (mozzarella), milk, lean meats and soy products. Pumpkin seeds contain the most tryptophan—576 mg/100 g. Although the tryptophan content of these products is high, they can be consumed without concern—it is not high enough to fear an excess of tryptophan in the body. However, tryptophan used as an ingredient in many supplements to combat sleep problems, prolonged stress and migraines, and to raise overall energy levels in the body may pose some risks. Increased urinary alkalinity facilitates indoxyl oxidation [199,200].
– Severe constipation also promotes bacterial overgrowth, thereby increasing the degradation of dietary tryptophan [30].

The factors that can lead to a change in the color of urine are presented in Table 1.

### 3.2. Changes in the Clarity and Transparency of Urine

**Figure 1:**
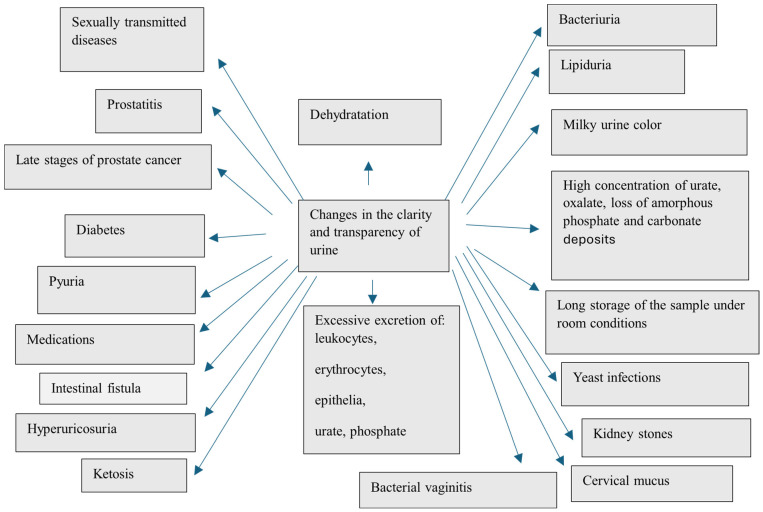
Factors that can change the clarity and transparency of urine [8,27,52,72,104,121,125,189,190,194,202,203,220,221,222,223,224,225,226,227,228,229,230,231,232,233,234,235,236,237,238,239,240,241].

Properly fresh urine is usually clear and transparent, without visible particles of suspended sediment. Opaque, cloudy urine that persists for several days has an unpleasant odor, indicative of a urinary tract infection or the presence of kidney stones [201] (Figure 1).

Possible causes of urine turbidity (Figure 1):

– Dehydration resulting in, among other things, the presence of an excessive concentration of urine’s aggregated and unaggregated elements. In addition, insufficient drinking of fluids can lead to the formation of stones in the bladder [22,72,121,125].
– Excretion of large leukocytes, erythrocytes, epithelia, urate and phosphate as a result of various pathological conditions [52,202,203].
– Bacteriuria (urinary tract/kidney infection) [104,204,205].
– Lipiduria—the presence of even trace amounts of lipids in the urine, caused mainly by chyluria [206] and trauma [207]. Lipiduria can also be caused by the presence of free cholesterol, cholesterol esters, triglycerides, free fatty acids and phospholipids [208,209,210,211]. In patients with low-grade proteinuria [211], lipid droplets are thought to originate from renal cysts containing degraded blood.
– Lactic staining of urine described earlier can also cause the interpretation of a cloudy sample [8,189,190,194].
– High concentrations of urate and oxalate, loss of amorphous phosphate and carbonate deposits (urine pH changes) [212,213,214].
– Long storage of the sample under room conditions [215,216,217].
– Yeast infections—*Candida albicans* in the vagina—resulting in the formation of a thick, cheesy, white discharge that mixes with urine [218,219].
– Kidney stones—cloudy urine is one of many symptoms—plus body pain (located in the side, back, below the ribs, groin or abdomen), frequent urination, fever and chills [52].
– Increased cervical mucus production during ovulation in women [220] may produce more malleable, milky or creamy mucus that mixes with urine to give it a cloudy appearance. This is considered a normal occurrence, but if the discharge has an unpleasant odor or a different color, a doctor should be consulted.
– Bacterial vaginitis—appearance of a grayish-white discharge that can mix with urine and make it cloudy [221,222].
– Sexually transmitted diseases such as gonorrhea and chlamydiosis can cause cloudy urine because they increase the production of a gray or white discharge in the vagina that can mix with urine [223,224,225].
– Prostatitis. One of the symptoms of this disease caused by a bacterial infection is discharge from the urethra, which can mix with urine and cause it to become cloudy. Benign prostatic growth, on the other hand, can make it difficult to empty the bladder and result in excessive bladder retention and a tendency to form stones [52,226].
– Late stages of prostate cancer—blood or sediment may accumulate in the obstructed bladder. If these are excreted with the urine, they can cause it to become cloudy. Also, prostatectomy surgery can sometimes lead to cloudy urine [226,227,228]. This may be due to the use of a catheter during the healing process.
– Diabetes—cloudy urine can indicate type 1 or type 2 diabetes (plus other symptoms). Hyperglycemia can also lead to kidney disorders or increase the risk of urinary tract infections—both conditions can cause urine to become cloudy—as well as damage blood vessels in the kidneys, leading to their malfunction. In addition, high blood sugar levels can cause proteins to be present in the urine, which reduces surface tension and can make the urine foamy [229,230,231].
– Pyuria, or an elevated white blood cell count that can pass into the urine due to diseases such as urinary tract infections (UTIs), gonorrhea, interstitial cystitis, tuberculosis, pneumonia and kidney disease [204,232,233] and from taking medications such as diuretics, penicillin and other antibiotics, proton pump inhibitors (e.g., omeprazole) and non-steroidal anti-inflammatory drugs such as aspirin and ibuprofen [234,235,236].
– Intestinal fistula—refers to the presence of an opening between the bladder and the intestine. Intestinal fistulas usually occur due to diseases such as diverticulitis, Crohn’s disease or colon cancer and can cause symptoms such as cloudy urine and abdominal bloating [237].
– Hyperuricosuria, that is, a high concentration of uric acid in the urine, can develop in people who frequently consume high-purine foods such as anchovies, shellfish, sardines, offal, peas, spinach, asparagus, mushrooms, yeast and alcoholic beverages [238,239,240].
– Ketosis—a condition in which high concentrations of ketone bodies appear in the urine. Ketosis occurs when the body begins to use fatty acids instead of glucose for energy. Many factors can cause ketosis, including low-carbohydrate diets, eating disorders, digestive disorders, prolonged diarrhea or vomiting, high-intensity exercise and pregnancy. A high number of ketone bodies in the urine can make it lipemic in color, which can sometimes be interpreted as a cloudy coloration [241].

### 3.3. Changes in the Odor of Urine

**Figure 2:**
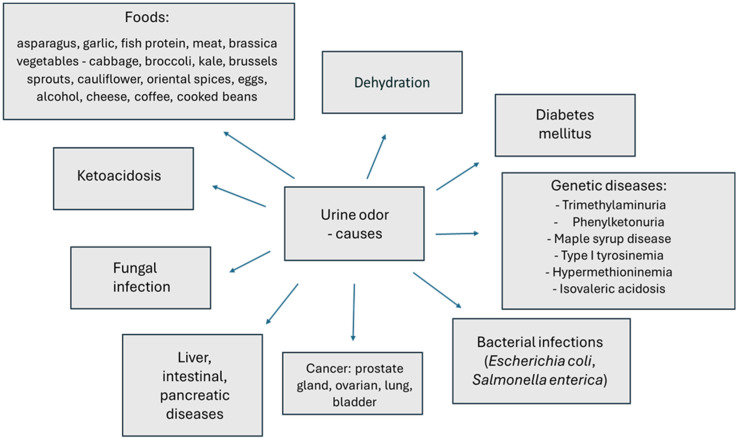
Factors that can change odor in urine [12,30,31,32,33,34,36,37,38,39,40,41,42,43,44,244,245,246,247,248,251,252,253,254,255,257].

The presence of an inappropriate urine odor is not always associated with the onset of a serious disease. In most cases, change in the odor of urine is the result of a specific diet or a lower tolerance to food products that contain, for example, a high concentration of sulfur. The presence of volatile organic compounds (VOCs) in urine is the result of intermediate or final metabolic transformations of various compounds [242]. In urine, they are often found in relatively higher concentrations, making them easier to detect [243]. Urine odor is highly variable, depending on diet, comorbidities, medications taken, and body condition, including, for example, dehydration (Figure 2).

– The urine of cancer patients contains volatile organic compounds (VOCs) produced specifically in response to a particular cancer, such as the prostate gland, [244,245] ovary, lung and bladder [246,247,248]. Serum prostate-specific antigen is the most commonly used biomarker for prostate cancer and urinary volatile organic compounds have been proposed as alternative biomarkers. Researchers have tried to determine whether certain VOCs in urine can cause a specific urine odor associated with cancer. These studies have confirmed the potential of using urine for diagnostic purposes. Recent work has also shown that characteristic VOC profiles in urine can be associated with infectious diseases [249,250].
– Foodstuffs containing substances whose metabolites can cause the appearance of a specific odor, e.g., asparagus (methyl mercaptan) [12,251,252], garlic (allyl mercaptan, allyl methyl sulfide (AMS), diallyl disulfide (DADS) and diallyl sulfoxide (DASO 2) [31,32], fish protein, meat [30,33], brassica vegetables—cabbage, broccoli, kale, brussels sprouts, cauliflower—oriental spices, eggs, alcohol, cheese, coffee or cooked beans or taking B vitamins [34,35].
– A state of dehydration and thus urine congestion or simple incontinence and involuntary urine leakage can cause a specific pungent odor [253].
– Fungal infection of the urinary tract—yeast-like odor [37].
– Diabetes mellitus causing urine to smell fruity indicates blood glucose levels are too high [36]. In contrast, ketoacidosis causes acetone odor (the smell of sour apples) and indicates diabetic ketoacidosis. Ketone bodies (of which acetone is the main representative) appear in the urine and cause metabolic acidosis, a state of disruption of the body’s acid–base balance [254].
– Liver, intestinal and pancreatic diseases cause a musty odor. Cases have been described with transient trimethylaminuria (TMA) associated with acute intestinal inflammation induced by dietary protein with urinary excretion of TMA and concurrent disease [34,38]. Another source of TMA in urine may be intestinal flora, mainly Anaerococcus, Providencia, Edwardsiella, Clostridium, Collinsella, Desulfovibrio, Lactobacillus and Proteus [251].
– Bacterial infections (Escherichia coli, Salmonella enterica), kidney stones, kidney disease—smell of ammonia, hydrogen sulfide, spoiled meat [34,255,256]. The main contributors to the production of the gaseous methanethiol that determines rotten odor are intestinal bacteria such as E. coli, Citrobacter and Proteus. Methanethiol is absorbed in the intestines then enters the blood and is excreted in the urine [257].
– Genetic diseases:
– Trimethylaminuria (trimethylaminemia) (also storing urine at high temperatures for too long) causes a fishy urine odor. The primary problem associated with trimethylaminuria is a deficiency in the enzyme FMO3 (flavin-containing monooxygenase). This enzyme is involved in the conversion of trimethylamine to trimethylamine oxide; when such conversions do not take place, trimethylamine is excreted from the body in, among other things, urine and sweat. The disease is inherited in an autosomal recessive manner (mutations in the genes encoding FMO3), which means that abnormal genes must be inherited from both parents in order to contract the disease [39].
– Phenylketonuria—mousey urine odor caused by this genetic metabolic disease, which involves the accumulation of one of the essential amino acids, phenylalanine, in excess in the body. Excessively high levels of phenylalanine in the blood lead to gradual depletion of the nervous system [40].
– Maple syrup disease—the smell of burnt sugar in medical terminology, also known as branched-chain amino acid ketoaciduria. This is a genetically determined metabolic disorder in which the body fails to break down the branched-chain amino acids leucine, isoleucine and valine (these are commonly found in foods, especially those rich in proteins) [4]. As a consequence, these substances and their toxic breakdown products (α-keto acids) accumulate in the body, leading to gradual poisoning and, if untreated, death. The cause of maple syrup disease is a deficiency in the activity of the enzyme complex of α-ketoacid dehydrogenases. It is caused by mutations in the BCKDHA, BCKDHB and DBT genes, which contain instructions on how to produce the aforementioned enzyme complex. The disease is inherited autosomal recessively. Alpha-keto acids and amino acids such as leucine, isoleucine and valine accumulate in the blood and urine (among others). The disease is diagnosed in the first days after a child’s birth [4,41].
– Type I tyrosinemia—the urine of patients gives off an odor of rancid butter or rotten mushrooms. In tyrosinemia, tyrosine is not properly metabolized as a result of the lack of fumarylacetoacetate hydrolase and tyrosine aminotransferase. The accumulation of excess tyrosine and toxic metabolites causes liver and kidney damage. Tyrosinemia is a disease inherited in an autosomal recessive manner [42].
– Hypermethioninemia is characterized by an excess of methionine in the blood of patients due to abnormal metabolism of this substance (mutations in the MAT1A, GNMT, or AHCY genes), as well as the smell of a cooked cabbage odor in their breath, sweat or urine. In many patients, hypermethioninemia may not produce visible symptoms; however, it can lead to tissue damage (e.g., liver, cerebellum) [34,43].
– Isovaleric acidosis is a rare genetic metabolic disorder caused by a mutation in the IVD gene. The disease is characterized by, among other things, a “sweaty feet” odor secreted in the urine [44]. The disorder is based on the body’s inability to break down leucine, due to reduced or absent activity of one of the enzymes, isovaleryl-CoA dehydrogenase. As a result, harmful metabolic products (organic acids, mainly isovaleric acid) accumulate in the body of the affected child, damaging the nervous system and internal organs and causing serious health problems. The symptoms of isovaleric acidosis can range in severity from mild to life-threatening.

### 3.4. Changes in the Specific Gravity of Urine

The specific gravity of urine (relative density of urine) depends on the amount of solutes and the amount of diuresis and renal tubular function (as well as kidney function, amount of fluids drunk, blood Ca and K concentrations, protein supply and sodium) [258,259,260]. In adults, normal urine weight ranges from 1.015–1.025 g/mL and is inversely proportional to fluid supply [258]. There are also data reporting that the specific gravity under physiological conditions is 1.001–1.035 g/mL. A large increase in the specific gravity of urine (>1.035 g/mL) may be due to excess of, among other things, glucose/protein/ethanol/radiological contrast (immediately after the examination)/excessive sweating/persistent vomiting/diarrhea and associated dehydration [47,52,73,112,113,114,115,116,259,261,262,263,264]. Each mg/dL of glucose in urine results in a 0.004 increase in urine specific gravity [262]. Each mg/dL of protein in urine results in a 0.003 increase in specific gravity and each 3 °C above the instrument calibration temperature results in a 0.001 increase in specific gravity [47,52,259,263].

The specific gravity of urine can increase due to the following:

– A diet containing vegetables and fruits rich in potassium (bananas, potatoes, dried figs, apples, apricots, leafy vegetables, but to a much greater extent supplementation with potassium preparations) [265]. High urinary potassium concentrations can also indicate two conditions: too much potassium in the serum (hyperkalemia) or too much loss of this element by the kidneys as a result of kidney damage or diseases that disrupt their function.
– Frequent consumption of meat, fish and dairy products [266,267].
– Dehydration, fever (excessive sweating), diarrhea, persistent vomiting, proteinuria, glucosuria, certain drugs (e.g., mannitol, dextran) and urinary excretion of certain exogenous substances (ethanol) [262,268].

Regardless of the degree of hydration of the body, consistent persistence of the relative density of urine at around 1.010–1.012 is a sign of isosthenuria and indicates a complete loss of the ability of the kidneys to thicken and dilute urine [269].

The specific gravity of urine is lowered by the following:

– Long-term low-protein diet [263].
– Hypotonic conductance, hypothermia, alkalosis (increase in plasma pH), renal tubular insufficiency, endocrine disorders (e.g., uremia) [27,126,270].
– Electrolyte disturbances—use of laxatives, frequent vomiting, use of diuretics (decrease in K, increase in serum Ca) [271,272].
– Kidney diseases [273].
– Adulteration of urine with various additives. This phenomenon can be considered both in vitro and in vivo [217,274,275]. In vivo adulteration occurs when a patient attempts to dilute the urine enough to bring the drug or drug concentrations below the detection limit, such as by drinking large amounts of fluids, herbal products or diuretics. For example, if the test shows creatinine values below the limit of quantification and a specific gravity of 1.004 g/mL, this indicates dilution of the urine [275]. In vitro adulteration, on the other hand, involves adding compounds to the urine sample that interfere with immunoassays or modify the structure of the molecule being tested so that it can no longer be detected [276,277,278,279,280]. Often used for this are vinegar, salt, bleach, fruit juices (grapefruit, lemon) or eye drops, and substances such as nitrites, hydrogen peroxide, pyridine or antiglutaric: glutaraldehyde [280,281]. The mechanisms of action of the aforementioned substances vary, depending on their physicochemical properties. For example, bleach has a direct effect on reagents and causes false-positive or false-negative results, depending on the immunoassay. Most adulterants are oxidants. They can react with, for example, a metabolite of tetrahydrocannabinol or with an antibody in an immunoassay, producing false-negative results in both the assay and gas chromatography–mass spectrometry (GC-MS) [280]. Glutaraldehyde is also used as a sterilizing or cleaning agent in hospitals, for example. It can cause false-negative results by reducing the optical density for detecting cannabis, amphetamines, methadone, opiates, cocaine and their metabolites by immunoassays. Finally, some adulterants can interfere with the extraction procedure itself [276].

### 3.5. Changes in the pH of the Urine

The urine reaction is a result of the diet used and the metabolic processes that take place in the kidneys, liver and lungs [217,282,283]. Urine pH ranges from 4.5 to 8; it is assumed that normal urine has a slightly acidic reaction, with pH = 5.5–6.0. Urine collected from the first morning micturition is usually slightly acidic (pH of about 6.5) [284]. The median pH of 24 h urine is about 6 [282]. This pH is the optimal for solubility of both uric acid and phosphate salts, which protect against the formation of stones in the urinary tract. At lower pH, the solubility of uric acid decreases, increasing the risk of crystals, while with an increase in pH above 6, the solubility of calcium phosphate decreases, increasing the risk of renal stones (as brushite and apatite_)_. Thus, urine pH is an important trigger for the formation of various types of stones in the urinary tract. The modern diet is characterized by the ubiquity of sodium chloride. Our ancestors fed mainly on plant foods rich in potassium-alkaline salts. This generates a lot of health problems resulting from the dissonance of genetic conditions and the mismatch between modern menus. 

A strongly acidic urine reaction can be caused by the following (among other things):

– Protein-rich diet (especially meat-rich) [283,285,286,287].
– Dehydration [22,110,114,115,116,122,261].
– Starvation [283,288].
– Systemic acidosis (ketoacidosis after ingestion of methanol or ethyl glycol), use of acidifying drugs, fever, some bacterial infections [283,289,290,291].

A strongly alkaline urine reaction can be caused by the following (among other things):

– A vegetarian diet with lots of fruits and vegetables (low in protein) and citrus juices. [283,285,286,292]
– Urinary tract infection with urease-containing bacteria (breakdown of urea to ammonia) [293,294].
– Long (>2.5 h) storage of urine samples → accelerated bacterial growth, increased breakdown of amino acids and urea [201,258,284].
– Use of certain medications (sodium bicarbonate, potassium citrate or acetazolamide) [295,296] kidney stones [292,297]. Bicarbonate intake, therefore, increases the buffering capacity of the body and has a strong alkalizing effect. Bicarbonate is a natural component of mineral water. A study of healthy subjects under standardized conditions showed a significant and sustained increase in urinary pH per day from 6.10 to 6.59 and citrate excretion from 3.045 to 4.554 mmol/24 h after consuming mineral water containing 3388 mg/L bicarbonate [292].

## 4. Conclusions

Testing a urine sample is usually the first stage in carrying out a general assessment before blood tests are needed. The composition of urine is variable and depends on a great many factors, such as the amounts and types of fluids ingested, food consumed and stimulants or drugs taken. It is essential to have knowledge of the factors that affect urine, including its color, clarity, odor and specific gravity. Well-standardized procedures for collecting, transporting, preparing and analyzing samples should become the basis for an effective diagnostic strategy in urinalysis. Although urinalysis is not recommended as a routine screening tool for the general population, in some cases it can alert the patients to abnormalities in the body and sensitize the medical staff for prompt/further medical action.

## Figures and Tables

**Table 1:** Factors that can change the color of urine.

Urine Colors	Factors
No color	overhydration [22,27,29]
	medications [23,24,25,26,27,28,130]
	cancers [27,133,134]
	renal disease [23,24,130,131,132,133]
	hypothyroidism [24]
	diabetes [27]
	excessive vasopressin [23,25,26]
Dark-yellow or amber	light dehydration/dehydration [22]
	working or being in a hot place [22]
	heavy physical exercise [108,109,110,111,112]
	severe diarrhea [55,73,117]
	laxatives [121,122,123,124,137]
	persistent vomiting [112,113,114,115,116,136]
	impaired kidney function [121,122,123,124]
Bright yellow	multivitamin supplements (B2, B12) [13,14,138]
Orange or red-orange	diet with lots of raw carrots [94]
	vitamin supplements (β-carotene) [94]
	laxatives [146]
	liver disorders [13]
	medications [146,187]
	*Citrobacter sedlaki* [188]
	crystalluria [139]
Black	iron supplementation [16,170]
	laxatives [171]
	melanoma [168]
	poisoning with nitrates (III/V), aniline dyes [170,172]
	alkaptonuria [173]
	medications [18,19,78,80,86,87,93,157,158,172,174,175]
	diet rich in black licorice [75]
Brown	rhubarb, broad beans, fava beans [77,165]
	hemoglobinuria, porphyria [21,148,153]
	copper poisoning [66]
	dehydrated [22,108,111]
	liver diseases [167]
	presence of melanocytes [101,168]
	extremely tough exercises [108,109,111,153]
	acetaminophen (overdose) [169]
Milky/white	electrolyte disorders (excess calcium, phosphate) [189]
	urinary tract infections (*E. Coli*, Proteus) [190,191,192]
	chyluria [8,193,194]
	presence of uric acid crystals (purine-rich foods: herring, anchovies, red meat) [195]
Reddish-pink or red	presence of urate [139]
	inflammation [139,140]
	foods rich in natural and added food dyes
	(beets, beet leaves, blackberries,
	blueberries, rhubarb, carrots) [17,75,76,94,146]
	menstrual blood [147]
	hemoglobinuria, porphyria [21,148,149,150,151,152,153,154]
	medications [18,19,78,87,88,97,155,156,157,158,159,160,161]
	lead or mercury poisoning [162,163]
	sample adulteration [164]
Purple	purple urinary pouch syndrome [196,197,198,199,200]
	tryptophan (supplements) [15]
	severe constipation [30]
Blue-green	foods rich in artificial dyes and foods like spinach and green asparagus [75]
	medications [84,85,92,176,177,178,179,180,181,182]
	dyes used intravenously in the diagnosis of kidney and bladder diseases [182,183]
	Drummond’s syndrome [184]
	familial hypercalcemia [185]
	prostatitis, bladder bacteremia (*Pseudomonas aeruginosa*) [89,186]
